# S7. KEYNOTE LECTURE: Immunobiological properties of the human cancer stem cells

**DOI:** 10.1186/2051-1426-2-S2-I4

**Published:** 2014-03-12

**Authors:** C Maccalli, EZ Elisabetta Zambon, FC Filippo Capocefalo, GP Giorgio Parmiani

**Affiliations:** 1 San Raffaele Scientific and University Institute, Unit of Immunbiotherapy of Melanoma and Solid Tumors, Department of Molecular Oncology, Milan, Italy

## Background

Cells with “stem cell properties”, denominated cancer stem cells (CSCs), have been recently isolated from a variety of human solid tumors. These cells have been considered as responsible of resistance to standard therapy such as chemotherapy and radiotherapy. Immunotherapy, due to its specificity and lack of toxicity can represent a promising approach to target cancer stem cells.

Thus, it is relevant to assess whether CSCs isolated from solid tumors, can be exploited as source of antigens to elicit T cell-mediated immune responses and to design novel immunotherapeutic protocols for tumors.

## Results

In our group CSCs from glioblastoma multiforme (GBM) and colorectal cancer (CRC) have been isolated from tumor biopsies and have been biologically characterized. Moreover, a detailed immunological characterization of these CSCs has been performed leading to the identification of a low immunogenic profile with negative immunoregulatory properties (*Di Tomaso et al*, *2010*).

We found that both CSCs and the differentiated counterpart of tumors (FBS tumor cells) expressed immune modulatory molecules, such as CTLA-4, PD-1, PDL-1 and B7H3, with, in some cases, higher levels in CSCS vs. FBS tumor cells. Furthermore, a differential gene signature that was confirmed at the protein level for some immunological-related molecules was also found for CSC and FBS lines. A candidate negative immunoregulatory molecule is represented by the indoleamine 2,3-dioxygenase (IDO), a molecule implicated in the generation of immune tolerance. By RT-PCR we detected the preferential increase of the mRNA of this molecule in CSCs vs FBS tumor cells following IFN-ϒ treatment. The functional activity of IDO determining, by a colorimetric assay, IDO-mediated tryptophan catabolism in culture supernatants was also found to be preferentially associated with CSCs.

Of note, CRC CICs expressed higher levels of IL-4 as compared with FBS tumor cell pairs. CIC-associated IL-4 could mediate, by cell-to cell contact, the inhibition of proliferation of T cells following the co-culture with autologous CIC.

The neutralization in functional assays of at least one of the negative modulatory signals mentioned above, led to the modulation of both the type and the quality of tumor-specific T cell immune responses.

Micro-RNA (miRNA) profile, including miRNA with immunoregulatory functions, has been identified to be specifically associated with CSCs vs. FBS tumor cell pairs as well.

## Conclusions

Multiple mechanisms of immunoregulation that are exploited by CSCs to escape from T cell-mediated surveillance have been identified. These results may allow designing more effective immunotherapy protocols to target CSCs from GBM and/or CRC patients.

